# Boron Neutron Capture Therapy for Recurrent Glioblastoma Multiforme: Imaging Evaluation of a Case With Long-Term Local Control and Survival

**DOI:** 10.7759/cureus.33898

**Published:** 2023-01-17

**Authors:** Shosei Shimizu, Kei Nakai, Yinuo Li, Masashi Mizumoto, Hiroaki Kumada, Eiichi Ishikawa, Tetsuya Yamamoto, Akira Matsumura, Hideyuki Sakurai

**Affiliations:** 1 Proton Beam Therapy Center, University of Tsukuba Hospital, Tsukuba, JPN; 2 Radiation Oncology, University of Tsukuba Hospital, Tsukuba, JPN; 3 Neurosurgery, University of Tsukuba Hospital, Tsukuba, JPN; 4 Radiation Oncology, Yokohama City University Hospital, Tsukuba, JPN; 5 Radiation Oncology, Tsukuba University, Tsukuba, JPN

**Keywords:** radiation therapy, adl (activities of daily living), glioblastoma, particle therapy, boron neutron capture therapy (bnct)

## Abstract

Glioblastoma (GBM) is difficult to cure with conventional multimodal treatment and has an extremely poor prognosis. Boron neutron capture therapy (BNCT) is a new particle therapy for malignant tumors in the brain and head and neck region. This radiotherapy utilizes a nuclear reaction between neutrons and a nonradioactive isotope, boron-10. In this method, a boron compound is administered transvenously into the body. The boron compound has the property of being selectively taken up only by the cells of malignant tumors, and the subsequent irradiation with neutrons can destroy malignant tumor cells without damaging normal cells. Since the irradiation dose to normal tissues is reduced in BNCT, it may be possible to re-irradiate malignant tumors that recur after radiotherapy. Clinical trials have reported prolonged survival and safety of BNCT in a small number of patients with refractory malignancies, including GBM, but these reports do not address quality of life or activities of daily living (ADL) after treatment, and there is no information on the assessment of local control by imaging. Here, we report a case of GBM that recurred after surgery, 60 Gy of conventional radiotherapy and standard treatment with temozolomide. The patient achieved long-term local control and survival over five years after BNCT and was able to maintain ADL at home without any specialist care. We describe the case with evaluation using longitudinal magnetic resonance imaging (MRI).

## Introduction

Glioblastoma multiforme (GBM) accounts for approximately 25-40% of central nervous system malignant tumors and is the most common primary malignant brain tumor [1,2]. This tumor is difficult to cure with conventional multimodality treatment and has an extremely poor prognosis. The median overall survival without treatment is less than three months, and the median survival with surgery alone is less than six months [3-5]. Because the invasive nature of the tumor makes total surgical resection difficult, the standard of care is minimally invasive surgery followed by radiotherapy and chemotherapy with temozolomide [6]. However, even with this treatment, median survival is poor at 14.6 months [7].

Boron neutron capture therapy (BNCT) is a new particle therapy for malignant tumors in the brain, head and neck regions. This radiotherapy utilizes a nuclear reaction between neutrons and a nonradioactive isotope, boron isotope (10B). In this method, a boron compound is administered transvenously into the body. The boron compound has the property of being selectively taken up only by the cells of malignant tumors, and the subsequent irradiation with neutrons can destroy malignant tumor cells without damaging normal cells. Since the total irradiation dose to normal tissues is reduced in BNCT, it may be possible to re-irradiate malignant tumors that recur after radiotherapy. Clinical studies have reported prolonged survival and safety of BNCT in a small number of patients with refractory malignancies, including GBM [8,9]. However, these reports do not mention the quality of life or activities of daily living (ADL) after treatment, and there is no information on the evaluation of local control using imaging. Here, we report a case of recurrent GBM after surgery, conventional radiotherapy of 60 Gy, and standard therapy with temozolomide, in which long-term local control and survival with the maintenance of ADL were achieved for more than five years after BNCT. We described the imaging evaluation of this case using magnetic resonance imaging (MRI).

## Case presentation

A 59-year-old man was diagnosed with a brain tumor in the left temporal lobe by head CT and MRI after the development of a speech disorder. The tumor was removed by craniotomy. The pathological diagnosis was GBM WHO grade IV. At the time, more than 10 years ago, genetic mutation tests for isocitrate dehydrogenase (IDH) and O^6^-methylguanine-DNA methyltransferase (MGMT) were not performed. Postoperatively, the patient received radiotherapy of 60 Gy/30 fractions and temozolomide (140 mg) for 42 days. The patient remained mildly aphasic but was able to communicate well and maintain ADLs at home. However, three months after surgery, local regrowth of the tumor was observed and recurrence was diagnosed. The patient was referred to our hospital and underwent BNCT with the locally recurrent tumor as the target volume. The patient had a fever for three days after irradiation as an acute adverse event, which resolved spontaneously. Although he was able to live at home without any medical or nursing assistance after the completion of the first surgery, one month after the BNCT, he was hospitalized for the need of assistance with walking. Subsequently, the patient suffered brain necrosis, for which surgical and medical intervention was required. Pathological diagnosis of the resected specimen revealed radiation necrosis. Bevacizumab was used for radiation brain necrosis. Four years and six months (54 months) later, the patient was diagnosed with recurrence, and after five years and two months (62 months), his general condition deteriorated due to the primary disease. Survival for this patient from the initial diagnosis was five years and eight months (68 months). The patient was able to live at home until death.

BNCT procedure

**Figure 1:**
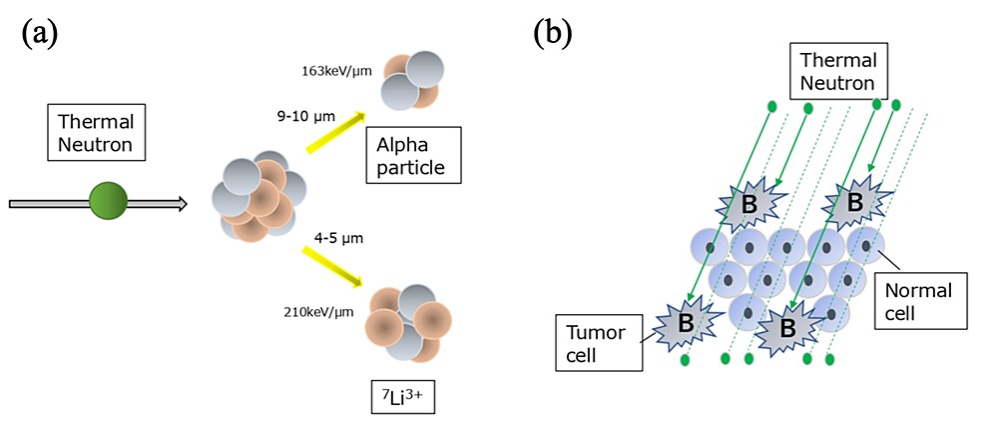
The reaction mechanism of BNCT. (a) Thermal neutrons react with 10B and generate two types of radiation particles, Alpha particles and lithium particles (7Li3+), both of which are less than 10 µm in radiation range. (b) 10B selectively accumulates in tumor cells without entering normal cells and reacts with thermal neutron, leaving normal cells undamaged. (Image Credits: SHOSEI SHIMIZU) BNCT: Boron neutron capture therapy

BNCT is an advanced type of radiotherapy, in which the radiation beam consists of two heavy particle beams produced by a reaction of a stable boron isotope (10B) with low-energy thermal neutrons. The reaction process is illustrated in Figure 1. 10B absorbs the thermal neutrons and immediately splits into Alpha particles and lithium particles (7Li3+) (Figure 1a). The radiation range of these nuclei is less than 10 μm, which is about the diameter of a cell, and thus, the radiobiological effects are limited to the area occupied by the boron compound. It implies that the side effects should be minimal for normal tissues based on the principle (Figure 1b). These two heavy ion beams are called high-LET beams and have a higher cell-killing effect than carbon ions used in heavy ion therapy.

**Figure 2:**
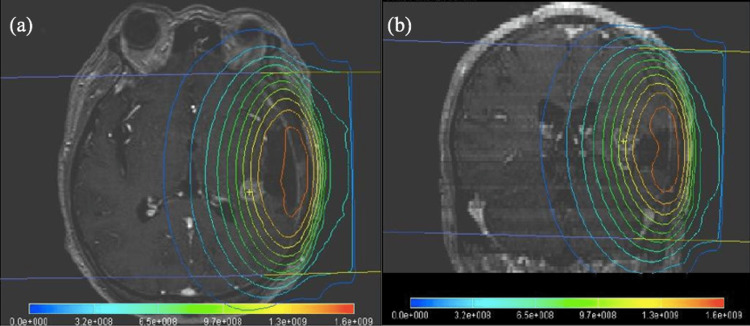
Dose distribution of BNCT for the recurrent GBM in the left temporal lobe on MRI. (a) Cross-sectional dose distribution diagram. (b) Coronal plane dose distribution diagram. BNCT: Boron neutron capture therapy; GBM: Glioblastoma

BNCT enables irradiation of tumor cells with thermal neutrons extracted from a nuclear reactor and is used clinically primarily for locally recurrent tumors after radiotherapy. In clinical practice, external irradiation of slightly higher energy epithermal neutrons is used to improve the depth of field. These epithermal neutrons repeatedly collide elastically with hydrogen nuclei in the body, losing energy and becoming thermal neutrons which react with 10B absorbed by tumor cells. In this case, the dose distribution during BNCT is shown in Figure 2. The fluence rate reached peaks at 1.5 to 2.5 cm from the body surface and rapidly decreased with depth, reaching about 1/10 of the peak at a depth of about 10 cm. This indicates that it is difficult to use BNCT for tumors located deep inside the body, as can be seen from the distribution of thermal neutrons after irradiation.

Boronophenylalanine (BPA) is often used as a boron compound in BNCT. In this study, PET (Positron Emission Tomography) diagnosis with 18F-fluoro-BPA (18F-BPA) was performed before BNCT to confirm the accumulation of BPA in tumor tissue, and BNCT was indicated when the accumulation was about three times greater than that in the surrounding normal brain. In this case, the dose was calculated in advance by simulating the maximum dose in the normal brain, as shown in Table 1. On the day of treatment, BPA at 250 mg/kg was administered one hour before the scheduled irradiation time, with administration terminated at the start of irradiation. The irradiation duration was 90 minutes. The average boron concentration in the blood was 14.0 ppm.

**Table 1:** Total relative biological effectiveness (RBE) dose of tumor and normal tissue. The normal tissue boron constant concentration was calculated as 12.6 μg/mL and the estimated tumor boron constant concentration as 42.8 μg/mL. Gross tumor volume (GTV) is regarded as the contrast-enhanced lesion, and clinical target volume (CTV) is the enlarged plus 2 cm area. The dose assumes that boron is evenly distributed in the tumor concentration there.

	Total Relative Biological Effectiveness (RBE) Dose (Gy-Eq)		
Target/Risk organ	Average dose (Gy)	Minimum dose (Gy)	Maximum dose (Gy)
CTV	41.2	11.0	61.7
GTV	43.9	18.0	60.4
Left brain	5.5	1.0	11.7
Right brain	1.2	0.4	3.3
Cerebellum	3.0	0.6	9.5
Brain stem	2.9	1.1	5.7
Left eye	3.0	1.1	5.7
Right eye	1.0	0.5	2.2
Cavernous sinus	2.8	1.4	4.6
Max skin dose			9.8

Imaging evaluation

**Figure 3:**
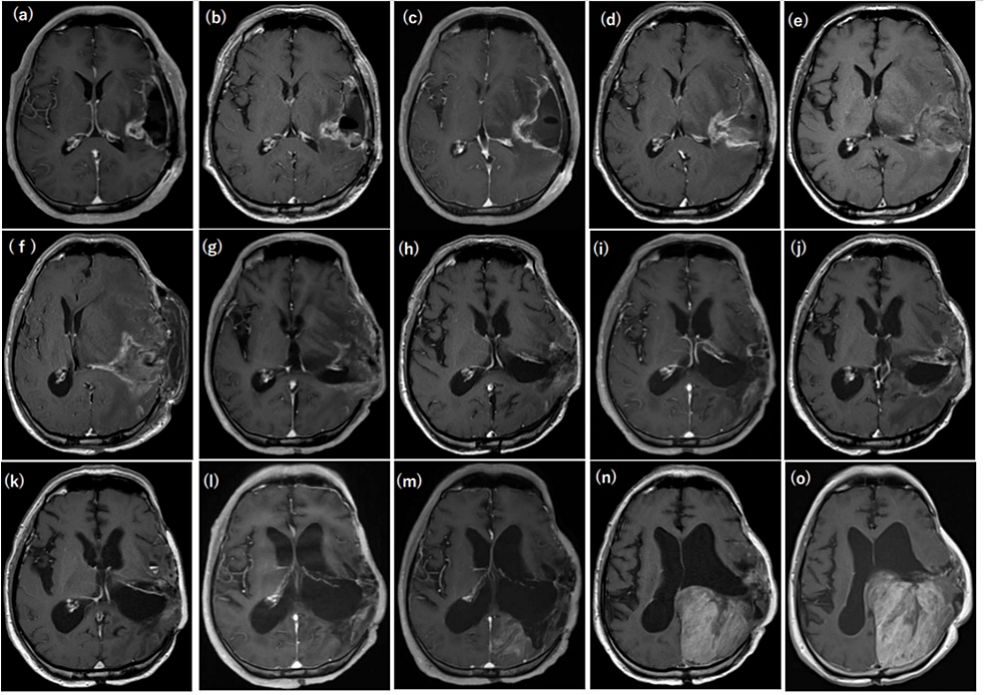
Contrast-enhanced T1-weighted axial brain MRI before, during, and after boron neutron capture therapy (BNCT). (a) One day before BNCT. (b) One week after BNCT. (c) One month after BNCT. (d) Three months after BNCT. (e) Six months after BNCT. Contrast-enhanced lesions appeared, but it was unclear whether these lesions were due to necrosis or recurrence. (f) Eight months after BNCT. The lesion was determined to be due to necrosis, and the necrotic lesion excision was performed. (g) 12 months after BNCT. (h) 18 months after BNCT. (i) 24 months after BNCT. (j) 30 months after BNCT. (k) 36 months after BNCT. (l) 48 months after BNCT. (m) 51 months after BNCT. A contrast-enhanced lesion appeared on the dorsal side of the irradiation field. (n) 54 months after BNCT. (o) 58 months after BNCT.

During the follow-up, the MRI scan was performed every 3-4 months, starting just before BNCT. The image immediately before BNCT showed a contrast-enhanced lesion at the surgical wound margin in the left temporal lobe in Figure 3a. On contrast-enhanced MRI one week after BNCT, the contrast effect for the surgical wound had increased in thickness, but it was difficult to discern from these images whether this was due to the treatment or the tumor (Figure 3b).

In contrast-enhanced MRI at one month after BNCT, the contrast-enhancement effect for the surgical wound appeared to be slightly reduced. And it was difficult to determine if the tumor was enlarged (Figure 3c). At three months after BNCT, there was no change in contrast effect or size around the surgical defect (Figure 3d). At six months, the contrast effect around the surgical defect was reduced and the margin of the defect showed a slightly higher signal than those insides, suggesting a mixture of hemorrhage and necrosis at the lesion (Figure 3e). At eight months, MRI revealed the elimination of necrotic lesion but it was not possible to determine whether the contrast effect around the defect was attributed to post-resection changes or the lesion itself. Cerebral edema and swelling had clearly exacerbated, possibly due to postoperative changes (Figure 3f). At 12 months after BNCT, as shown in Figure 3g, there was a significant decrease in the contrast effect around the defect compared to that at eight months, which indicates a good sign that the tumor was not aggravated. The brain midline shift also improved and it was concluded that there was no recurrence. Figure 3h demonstrates that 18 months after BNCT, the contrast effect at the limbus had also diminished, and edema and swelling in the cerebral hemispheres had resolved, again indicating no tumor recurrence.

Contrast-enhanced MRI at 24 months after BNCT showed a further weakening of the limbic contrast effect and improvement of edema and swelling in the cerebral hemispheres, with no evidence of tumor recurrence (Figure 3i). At 30 months after BNCT, there was no obvious change in the medial aspect of the original wound, but some hyperintense signals were seen dorsally and laterally, making it difficult to determine whether the tumor had recurred (Figure 3j). At 36 months after BNCT in Figure 3k, there was a diminished contrast-enhancement surrounding the defect and improvement of edema and swelling of the cerebral hemispheres compared to the six-month earlier images. At 48 months, a heterogeneous contrast effect was observed in Figure 3l spreading to the superficial side of the defect site with a mixture of low and high signals, suggesting the lesion that was radiation necrosis rather than tumor recurrence. At 51 months, a heterogeneous area of radiation necrosis became apparent on the dorsal surface (left parietal to occipital lobes). Although the possibility of tumor recurrence was considered in the differential diagnosis, the spread of necrosis in the dorsal region was ultimately identified as the determining factor at that time (Figure 3m).

Contrast-enhanced MRI at 54 months after BNCT (Figure 3n) showed that the dorsal heterogeneous contrast-enhanced lesion continued to increase in size to 45×49×53 mm with mixed radiation necrosis. It was strongly suspected to be a recurrence although there was no associated ventricular enlargement or seeding. And eventually, the lesion was confirmed to be a local recurrence. The last MRI of the patient was performed at month 58 after BNCT (Figure 3o), and the image revealed a coarse, substantial mass in the parietal to the occipital region that had increased in size markedly in a short period of time. The interior of the mass showed a brush-like overall pale contrast effect, and encompassed vascular structures along the white matter fibers on the head side, extending from the subcortical white matter to the cortex of the left parietal lobe. Such a rapid increase in radiation necrosis after about five years of radiation therapy is rare and it was eventually identified as a mixture of necrosis and tumor recurrence.

## Discussion

Surgical resection is the most effective treatment for benign tumors and low-grade gliomas, but GBMs are difficult to resect because of their invasive growth [10]. In addition, these tumors progress rapidly. The standard treatment is maximal-safe resection, followed by temozolomide and radiotherapy in 60 Gy/30 fractions [6]. In general, the prognosis is very poor with a median survival of 14.6 months [6]. Long-term survival is unlikely, with 90% of patients relapsing within two years after standard therapy, and reoperation, reirradiation, and further drug therapy are rarely effective [7]. Park et al. classified cases with reoperation after recurrence into good, intermediate, and poor prognoses based on the general condition, tumor size, age, and time to recurrence, and examined survival after reoperation [11]. The median survival times were 10.8, 4.5, and 1.0 months, respectively, and the survival time of patients judged to have a good prognosis exceeded 10 months [11]. Regarding reirradiation after recurrence, Combs et al. reported a median survival of 10 months, but the indication is limited to patients with good general conditions and small tumors [12].

The present patient had a relatively large tumor and was classified as intermediate-risk [11]. Thus, the efficacy of treatment with reoperation and reirradiation at the time of recurrence was expected to be poor. This situation led us to consider BNCT as an alternative to surgery, conventional radiotherapy, and chemotherapy at the time of recurrence. The approval of commercially available accelerator-based neutron sources and boron products in Japan has made it possible to treat a variety of superficial refractory tumors. In particular, this has opened the possibility of BNCT for refractory tumors that have relapsed after standard treatment [8].

In the use of BNCT for brain tumors, Kageji et al. reported a median survival time (MST) of 19.5 months for 23 patients with first-episode glioblastoma treated with BNCT alone [13]. The results of a study on BNCT for primary GBM indicated that there was no significant difference in time to tumor progression (TTP) between the intraoperative BNCT group (TTP: 12.0 months) and the external irradiation BNCT group (TTP: 11.9 months). The median TTP for all patients was 25.7 months. Only one grade 4 case presented with brain swelling requiring surgery, and there was no other grade 3 or higher adverse events. BNCT for first-occurrence GBM resulted in a good survival rate with an acceptable rate of adverse events [9]. Miyatake et al. obtained an MST of 9.6 months after BNCT in 19 patients with recurrent GBM [14], and a study in Sweden of BNCT in 12 patients with recurrent GBM found an MST of 8.7 months after BNCT and an MST from initial diagnosis of 22.2 months [15]. The outcomes of BNCT are shown in Table 2. These reports suggest that BNCT is an effective treatment modality after recurrence, but potential adverse events must be monitored carefully. Radiotherapy for GBM is the most important adjuvant therapy to enhance the curative effect. Based on the results of several large clinical trials, a total dose of 60 Gy/30 fractions is considered standard [16,17]. Even with local irradiation, the tolerated dose to the brain is 60 Gy with TD5/5 [18]. The prognosis for conventional GBM is generally short and consideration of late effects is often not a priority; however, the expected long-term survival with BNCT makes periodic imaging and follow-up necessary to detect brain necrosis, which can occur with reirradiation. That is, dermatitis and brain necrosis due to reirradiation are common problems in BNCT, as a cell-selective treatment, as well as in conventional radiotherapy. Empiric treatments for brain necrosis include the administration of steroids and anticoagulants and surgical resection of brain tissue. Development of radiation-induced brain necrosis is thought to be due in part to increased vascular permeability and edema as a result of abnormal angiogenesis caused by overproduction of intracellular vascular endothelial growth factor (VEGF), and the molecularly targeted drug bevacizumab has been found to be effective against this necrosis [19,20]. In our patient, bevacizumab was started postoperatively and was continued until the end of treatment. Previous reports have shown that brain necrosis appears in less than 12 months after radiotherapy followed by BNCT [19,20]. In our case, brain necrosis appeared at one month and eight months after BNCT, but thereafter, there was no obvious necrosis on imaging. In general, patients with GBM have difficulty in daily life due to the tumor and treatment [21-23]. However, our patient was able to maintain ADLs for a long period of time and was able to perform daily activities at home. BNCT at recurrence enabled local control for four years and six months (54 months), and the patient was able to live at home for five years and eight months (68 months) after the initial diagnosis. Recently, long-term survival has been reported for patients with IDH mutations in combination with treatment such as proton beam therapy and immunotherapy [24,25]. Survival after BNCT for recurrent GBM, in this case, was longer than that in previous BNCT cases, with good local control and no evidence of recurrence on imaging for a long period. The maintenance of ADLs that allowed the patient to live at home after recurrence and the safety of BNCT are key aspects of this case report.

**Table 2:** Treatment results of BNCT for GBM. TTP: Time to tumor progression; MST: Median survival time; OS: Overall survival; BNCT: Boron neutron capture therapy; GBM: Glioblastoma.

Authors	Year	Number of patients	Intervention	Tumor type	Outcome	Reference No.
Yamamoto et al.	2009	15	Operation+Intraoperative NCT (n=7) BNCT+ Photon radiation therapy (n=8)	primary	TTP: 12.0 months (M) TTP: 11.9 M	[9]
Kageji et al.	2014	23	BNCT alone	primary	MST: 19.5 M 2-year OS: 31.8%	[13]
Miyatake et al.	2009	19 (Malignant glioma=22)	Operation+BNCT(n=12) Temozolomide+BNCT(n=10)	recurrent	MST: 9.6 M (after BNCT)	[14]
Pellettieri et al.	2008	12	Temozolomide+BNCT	recurrent	MST: 8.7 M (after BNCT)	[15]

## Conclusions

This case suggests that tumor control and maintenance of ADLs in long-term survival after recurrent GBM are possible with BNCT if adverse events of radiation dermatitis and brain necrosis can be overcome.
